# Comprehensive *in vivo* and *in vitro* metabolic profiling of amphenmulin: a novel pleuromutilin derivative characterized by UHPLC-Q-TOF-MS/MS

**DOI:** 10.3389/fvets.2025.1660872

**Published:** 2025-08-21

**Authors:** Wenxiang Wang, Luoju Wang, Qin Deng, Dehai Su, Youzhi Tang, Huanzhong Ding

**Affiliations:** ^1^Guangdong Key Laboratory for Veterinary Drug Development and Safety Evaluation, College of Veterinary Medicine, South China Agricultural University, Guangzhou, China; ^2^Shandong Qilu King-Phar Pharmaceutical Co. Ltd, Jinan, China

**Keywords:** pleuromutilin, UHPLC-Q/TOF, metabolites, liver microsomes, *in vivo*

## Abstract

Amphenmulin is a novel pleuromutilin derivative with proven excellent antibacterial activity. To investigate its metabolism in animals, ultra-high-performance liquid chromatography coupled with quadrupole/time-of-flight mass spectrometry (UHPLC-Q-TOF-MS/MS) was employed to analyze and identify *in vivo* metabolites in rats and chickens and *in vitro* using human, rat, pig, chicken and beagle dog liver microsomes. We identified 18 metabolites from liver microsomes and 24 and 17 *in vivo* metabolites for rats and chickens, respectively. The primary amphenmulin metabolic pathways involved sulfur oxidation of the side chain, hydroxylations on the aniline moiety and the parent mutilin nucleus, and the primary *in vivo* metabolites in rats and chickens were amphenmulin-sulfoxide (M2) and hydroxy-amphenmulin (M1). These findings provide a structural basis for further investigation into the pharmacokinetics and safety profile of amphenmulin and serve as a reference for the development and optimization of other pleuromutilin derivatives.

## Introduction

Pleuromutilin was a natural product antibiotic first isolated from the basiomycetes *Clitopilus scyphoides* and *Pleurotus passeckeranius* (1). This compound is translational inhibitor that tightly bind within the peptidyl transferase center on the 50S ribosomal subunit (2). This unique mechanism of action minimizes the emergence of resistant strains and has no cross-resistance with other protein synthesis inhibitors (3, 4). Currently, an increasing number of semi-synthetic pleuromutilin derivatives are being developed and investigated, expanding their antibacterial spectrum to include methicillin-resistant *Staphylococcus aureus* (MRSA), *Mycoplasma* spp., *Chlamydia* spp. and anaerobes such as *Clostridium* spp. (5–7). The pleuromutilin family demonstrates significant potential in the development and optimization of new antibiotics to combat the growing complexity of bacterial infections.

**Figure 1:**
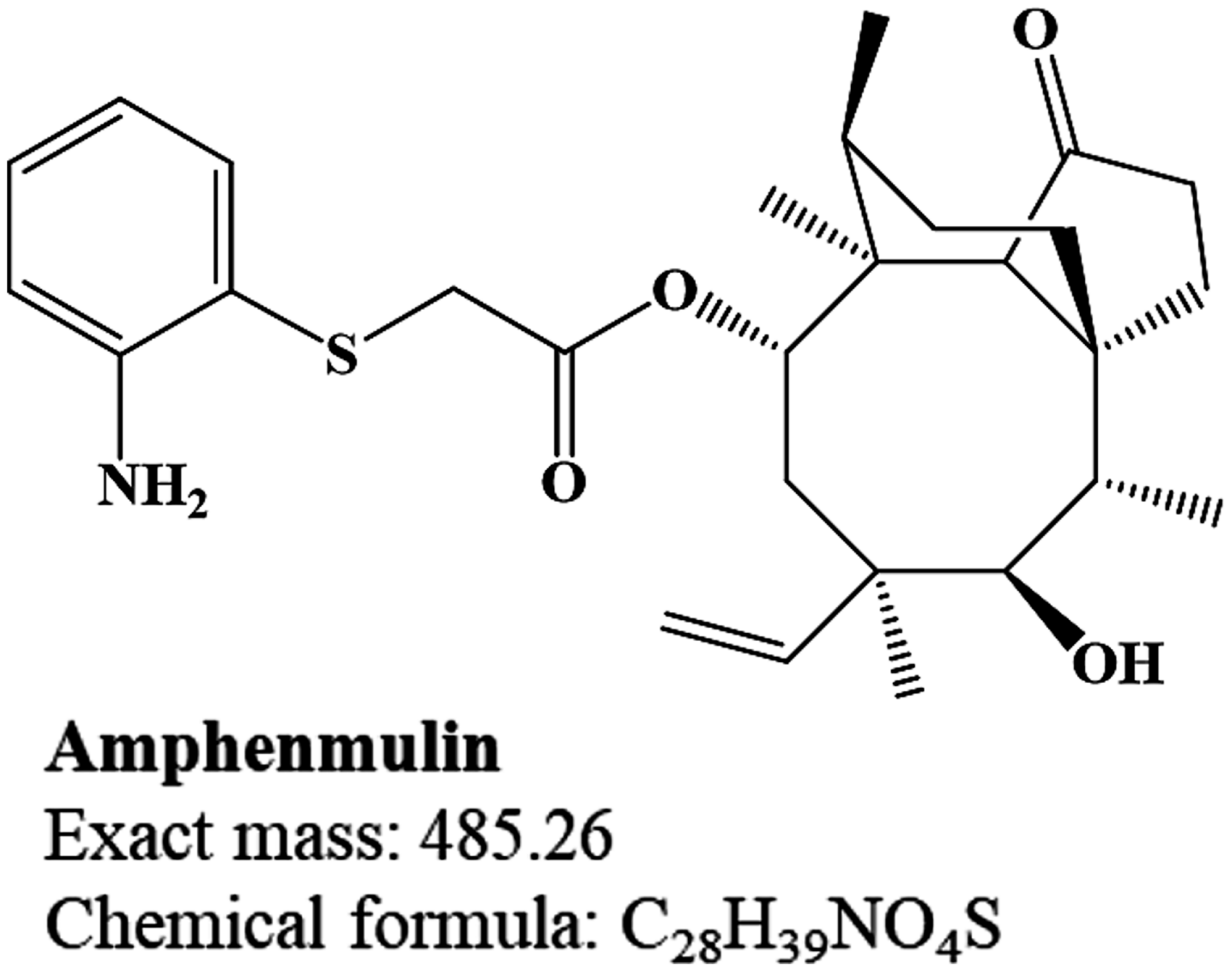
Chemical structure of amphenmulin (APM).

However, only a few pleuromutilin derivatives have successfully progressed to clinical applications and these include tiamulin and valnemulin for veterinary use, retapamulin for skin infections and lefamulin for human bacterial pneumonia (8). The tricyclic mutilin nucleus of pleuromutilin is crucial for its biological activity and almost all structural modifications and decorations of pleuromutilin derivatives focus on the *C*14 side chain (9, 10). This substituent is significantly linked to the antibacterial activity, metabolism and pharmacokinetic (PK) characteristics of the entire compound (5, 11–13). In addition, a basic sulfide center has also been modified to improve the activity of these pleuromutilin derivatives and all marketed derivatives retain this thioether structure (7, 14). Building on this feature, a series of pleuromutilin derivatives were designed and synthesized, among which amphenmulin (APM), 22-(2-amino-phenylsulfanyl)-22-deoxypleuromutilin (Figure 1), was identified as the lead compound for its robust antibacterial activity and was selected for further investigation (15).

APM demonstrated superior antibacterial activity against *S. aureus* compared to tiamulin and showed actual non-toxicity in acute toxicity tests on mice (16). We further elucidated the PK characteristics of APM in chickens and established a pharmacokinetic/pharmacodynamic (PK/PD) simultaneous model against *Mycoplasma gallisepticum* (17). However, drug metabolism is a crucial part of the early evaluation of new drugs since some metabolites may possess additional pharmacological activity, reactivity or toxicity (18, 19) and pose potential drug residue risks in food animals (20). We therefore sought to clarify the metabolic characteristics of APM in anticipation of providing data support for its pharmaceutical development.

Several metabolic studies of pleuromutilin derivatives have been reported. For instance, the primary metabolite of lefamulin is *2R-OH* lefamulin (21, 22). In addition, the primary metabolic pathways for tiamulin are N-deethylation and hydroxylation reactions (8α, 2β positions) (23, 24). Berner et al. (25) also proposed the hydroxylation positions of 1β, 2β and 8α in the mutilin part of pleuromutilin. In addition, valnemulin metabolism is primarily mutilin hydroxylation, *S*-oxidation of the side chain and hydrolysis of the amide bond (26). APM has the same nucleus structure with several reported pleuromutilin derivatives and the metabolic characteristics of these similar drugs can provide important references for the study of APM.

Quadrupole time-of-flight mass spectrometry is recognized for its rapid data acquisition, high resolution, and high sensitivity, making it an excellent choice for the identification and analysis of metabolites (27, 28). In this study, we conducted a combined *in vitro* investigation of APM metabolism in liver microsomes of different species, as well as *in vivo* metabolism studies in rats and chickens, utilizing ultra-high-performance liquid chromatography coupled with quadrupole time-of-flight mass spectrometry (UHPLC-Q-TOF-MS/MS). The precise information on parent and product ions were then utilized to infer the elemental composition and chemical structure of metabolites to thereby elucidate the pathways of APM metabolism.

## Materials and methods

### Chemicals and reagents

Amphenmulin (>99%) was synthesized by our laboratory according to the published methods and identified by nuclear magnetic resonance spectroscopy and high-resolution mass spectrometry (15). Reduced nicotinamide adenine dinucleotide phosphate (NADPH), uridine 5′-diphosphate glucuronic acid (UDPGA), alamethicin (250 μg/mL), D-glucaro-1,4-lactone (50 mM) and liver microsomes (20 mg/mL) from different species were all purchased from IPHASE BIOSCIENCES (Suzhou, China). Testosterone (98%), phosphate-buffered saline (PBS) and tris–HCl buffer were procured from Solarbio Life Sciences (Beijing, China). Ethyl acetate and hexane were analytical grade, procured from Macklin Biochemical Technology (Shanghai, China). Acetonitrile, methanol, and formic acid, all of LC/MS grade, were obtained from Thermo Fisher Scientific (Waltham, MA). Water was prepared using a Milli-Q water purification system (Millipore, Billerica, MA).

### Animals

Five-week-old Sprague–Dawley (SD) rats (three male and three female, body weight range 145–169 g), purchased from the Guangdong Medical Laboratory Animal Center. Adult yellow-feathered broiler chickens (three male and three female, body weight range 1.36–1.78 kg) were obtained from the Guangdong Academy of Agricultural Sciences. All animals were kept under standard conditions, fed with standard diets lacking drugs and watered *ad libitum*. All experimental procedures were approved by the Animal Ethics Committee of South China Agricultural University (approval number: 2024c055).

### SMARTCyp prediction of APM metabolic sites

The SMARTCyp online tool^1^ is based on quantum chemical methods for calculating the activation energy of cytochrome P-450 (CYP) reactions with ligand molecular fragments. The level of activation energy has been used to identify metabolically labile sites *in silico* (29–31) and to predict the potential metabolic sites of APM in cytochrome P450-mediated reactions.

### Incubation of APM with liver microsomes

APM was incubated with human, rat, pig, chicken, and beagle dog liver microsomes under identical conditions in a volume of 200 μL. The phase I incubation system consisted of 0.1 M phosphate buffer pH 7.4, NADPH and 0.5 mg/mL liver microsomal protein. Phase II consisted of Tris–HCl buffer pH 7.4, UDPGA, and 0.5 mg/mL liver microsomal protein, alamethicin was additionally included to enhance the enzymatic activity and efficiency of glucuronosyltransferase, and D-glucaro-1,4-lactone was used as a *β*-glucuronidase inhibitor. The reaction mixtures were pre-incubated at 37 °C for 5 min and APM was then added and allowed to react for 1 h. The reactions were terminated by addition of pre-cooled acetonitrile (200 μL) and vortex-mixed and centrifuged at 12000 rpm for 10 min at 4 °C. The supernatant was filtered through a 0.22 μm membrane for UHPLC-Q/TOF analysis. All experiments were conducted in triplicate with negative controls consisting of samples without the substrate and NADPH/UDPGA, and testosterone was used as a positive control substrate in place of APM. The formation of 6β-hydroxy testosterone was monitored to confirm the metabolic activity of the microsomal preparations.

### *In vivo* metabolism of APM in rats and chickens

Six SD rats and six yellow-feathered broiler chickens were housed individually in metabolic cages and given a single dose of 10 mg/kg APM via intravenous injection. Feces and urine samples from each animal were collected at 12 and 24 h after administration, and the two time points samples were pooled and thoroughly mixed prior to extraction. Blood samples were collected at 30 min, 2, 6 and 12 h following APM administration. Plasma was separated by centrifugation from blood samples using standard procedures and mixed with acetonitrile (1:4), vortexed and centrifuged at 10,000 rpm for 10 min at 4 °C. The supernatant was then filtered through a 0.22 μm membrane for analysis.

Urine samples were processed using 1 mL urine placed in a 15 mL centrifuge tubes containing 5 mL of ethyl acetate as the extraction solvent. After vortexing and mixing for 5 min, the mixture was centrifuged at 10,000 rpm for 10 min at 4 °C. The supernatant was transferred to a new centrifuge tube and the extraction was repeated once more. The combined supernatants were concentrated under a stream of nitrogen at 40 °C and the residue was redissolved in 1.0 mL of acetonitrile/water (6:4, v/v) and then treated with 2 mL of n-hexane. The lower solution was collected and centrifuged at 4 °C at 10,000 rpm for 5 min, then filtered through a 0.22 μm membrane for UHPLC-Q/TOF analysis.

Fecal samples were processed using 0.5 g of homogenized feces and the same method as for the urine samples. The resulting solution was then added to an OASIS HLB solid-phase extraction cartridge (Waters, Milford, MA) that had been previously activated with 3 mL of methanol and equilibrated with 3 mL of water. The cartridge was washed with 3 mL water followed by elution with 3 mL acetonitrile. Finally, 1 mL of the collected eluate was filtered through a 0.22 μm membrane for UHPLC-Q/TOF analysis.

### Instrumentation and conditions

Chromatographic separation of APM metabolites in all samples was conducted on an ACQUITY UHPLC system (Waters) equipped with an Acquity BEH RP18 column (50 mm × 2.1 mm, 1.7 μm). The mobile phases consisted of 0.1% formic acid in water (A) and acetonitrile (B) using a flow rate of 0.3 mL/min and an injection volume of 2 μL. The gradient elution program was as follows: 0 min, 5% B; 2 min, 5% B; 16 min, 100% B; 17 min, 100% B; 18 min, 5% B; 20 min, 5% B.

Mass spectrometric analysis was performed using the XEVO G2-XS QTOF (Waters) with electrospray ionization in positive ion mode (ESI^+^) for MS^E^ data acquisition. The main mass spectrometry parameters were as follows: mass scan range, 100–800 Da; desolvation temperature, 350 °C; desolvation gas, 650 L/h; capillary, 3 kV; source temperature, 120 °C; cone gas, 50 L/h; cone voltage, 24 V; collision energy was set at 5 V for low energy and 15 to 40 V for high energy. Throughout the scan, 200 ng/mL leucine enkephalin served as the tuning reference substance and was infused at a flow rate of 10 μL/min and possessed a characteristic ion *m/z* 556.2771 that was monitored for real-time mass spectrometry calibration.

### Data processing

Comparative analysis of UHPLC-Q/TOF samples was performed based on the obtained accurate ion masses and extracted ion chromatograms (EICs) using UNIFI Scientific Information System (Waters). To improve the accuracy, the mass defect filter threshold was set to 50 mDa, the exact mass error was kept ≤5 ppm and the retention time error was within 0.5 min. This process facilitated the screening and identification of all potential metabolites of APM to infer their elemental compositions. In addition, orthogonal partial least squares-discriminant analysis (OPLS-DA) was performed using SIMCA-14.1 software (Umetrics, Kinnelon, NJ). Metabolite abundance data from different species were pareto-scaled before analysis to enhance group separation while minimizing noise. The resulting score plots and loading scatter plots were used to visualize species-specific metabolic distribution and contribution of individual metabolites.

## Results and discussion

### Ionization and fragmentation pathways of APM

**Figure 2:**
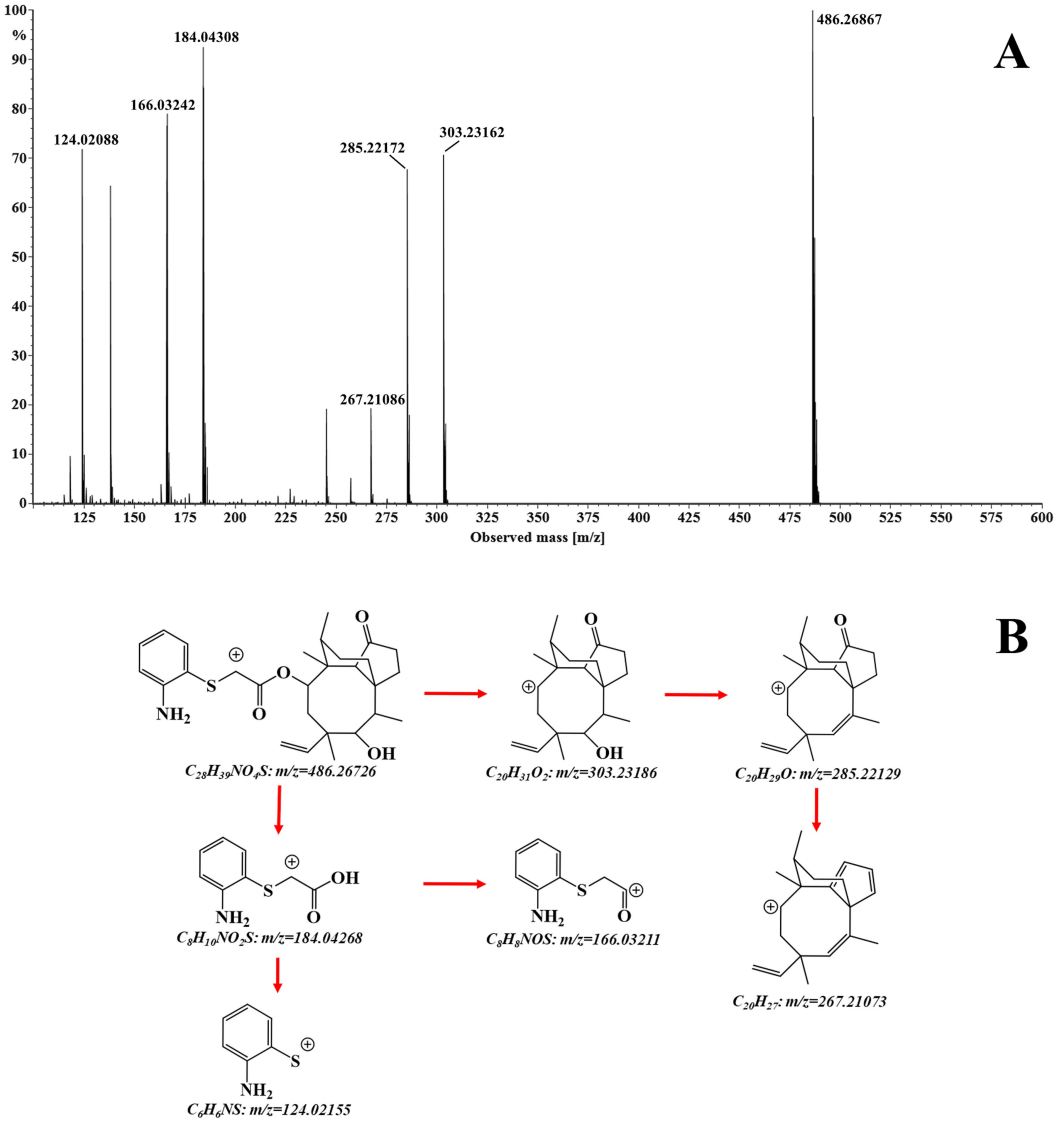
**(A)** Accurate MS/MS spectrum and **(B)** fragmentation pathways of APM.

The structural assignment of APM and its fragment ions was performed using the elemental composition calculator in UNIFI (Table 1). The measured mass values of the ions deviated from the theoretical values by −0.6 to 1.4 mDa and were all within a range of ±5 ppm. This indicated high mass accuracy of the instrument that would serve to ensure the reliability of the compound and fragment ion assignments. For APM, the MS/MS spectrum (Figure 2A) under positive ion mode indicated a protonated molecular ion [M + H]^+^ (C_28_H_39_NO_4_S^+^, *m/z* 486.2673). This parent ion underwent cleavage of the ester bond that yielded complementary product ions at *m/z* 184.0427 and *m/z* 303.2319. Fragmentation of the ion at *m/z* 184.0427 involved the breaking of the sulfide and ester bonds to generate fragment ions at *m/z* 166.0321 and *m/z* 124.0215. The *m/z* 303.2319 ion lost one and two H_2_O molecules to form product ions at *m/z* 285.2213 and *m/z* 267.2107, respectively (Figure 2B). These characteristic fragment ions serve as crucial evidence for the subsequent identification of APM metabolites.

**Table 1:** Elemental composition, measured masses, theoretical masses, and mass error of major APM ions.

Elemental composition [M + H]^+^	Theoretical mass (Da)	Measured mass (Da)	Mass error (mDa)	Mass error (ppm)
C_28_H_39_NO_4_S^+^	486.2673	486.2687	1.4	2.9
C_20_H_31_O_2_^+^	303.2319	303.2316	−0.2	−0.8
C_8_H_10_NO_2_S^+^	184.0427	184.0431	0.4	2.2
C_20_H_29_O^+^	285.2213	285.2217	0.4	1.5
C_20_H_27_^+^	267.2107	267.2109	0.1	0.5
C_8_H_8_NOS^+^	166.0321	166.0324	0.3	1.9
C_6_H_6_NS^+^	124.0215	124.0209	−0.6	−4.8

### Identification of APM metabolites *in vivo* and *in vitro*

**Figure 3:**
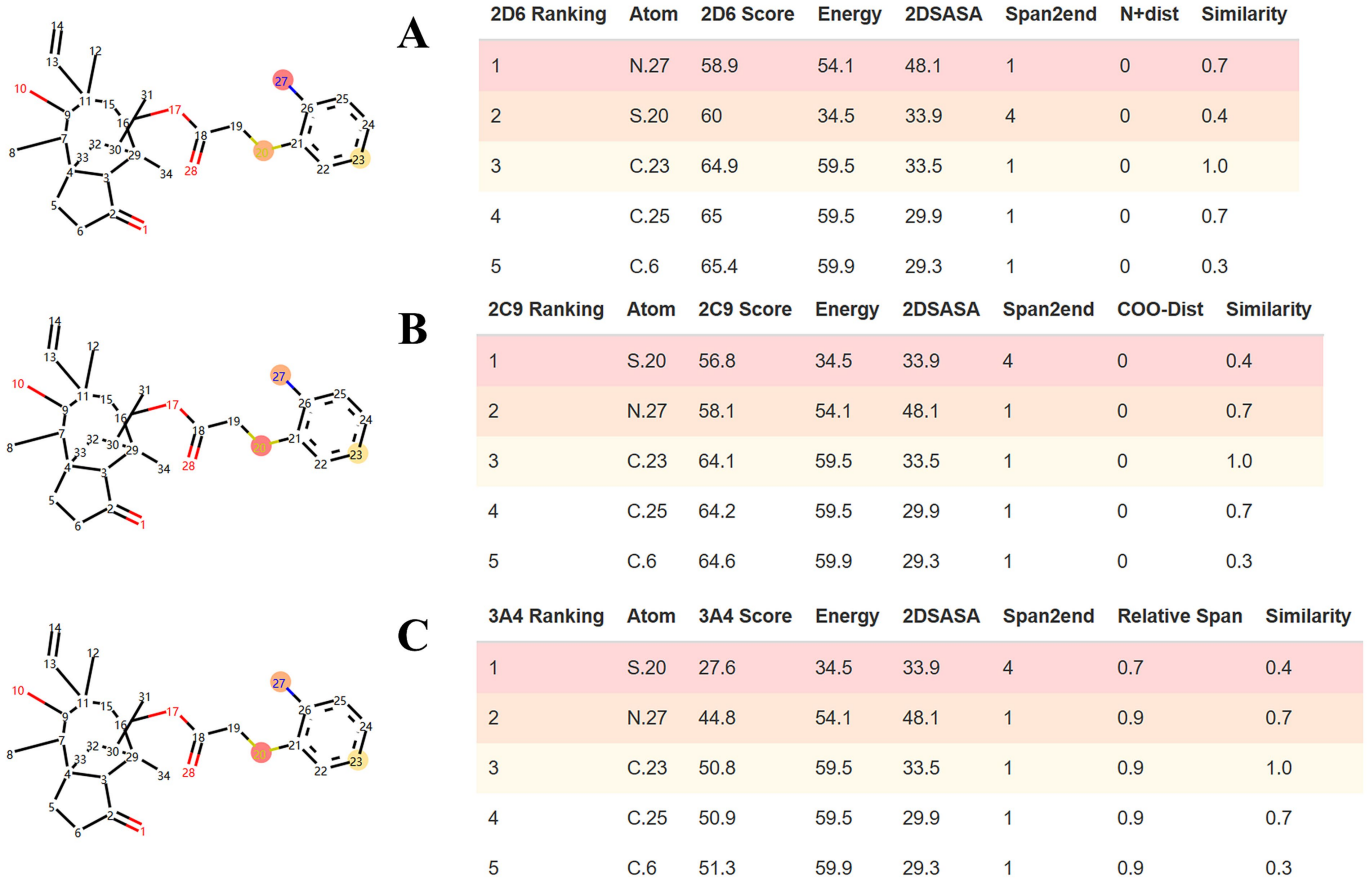
SMARTCyp predictions of APM metabolic sites for **(A)** CYP2D6, **(B)** CYP2C9, and **(C)** CYP3A4.

SMARTCyp was employed to predict the sites of metabolism (SOM) within the APM structure. As shown in Figure 3, the standard algorithms for CYP3A4, CYP2D6 and CYP2C9 identified the sulfur in the side chain, and the aniline moiety (amino nitrogen and the para-carbon) as key potential metabolic sites for APM.

**Figure 4:**
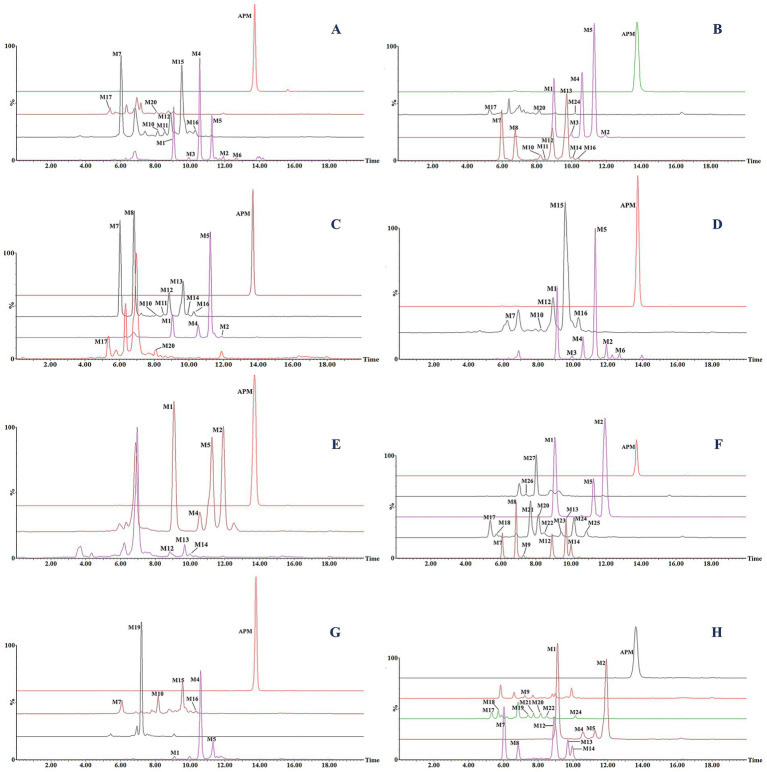
Extracted ion chromatograms (EICs) of APM and its metabolites in liver microsome incubation systems for **(A)** rat, **(B)** human, **(C)** pig, **(D)** dog and **(E)** chicken. EICs of *in vivo* metabolites from **(F)** rat feces, **(G)** rat urine, and **(H)** chicken feces.

**Figure 5:**
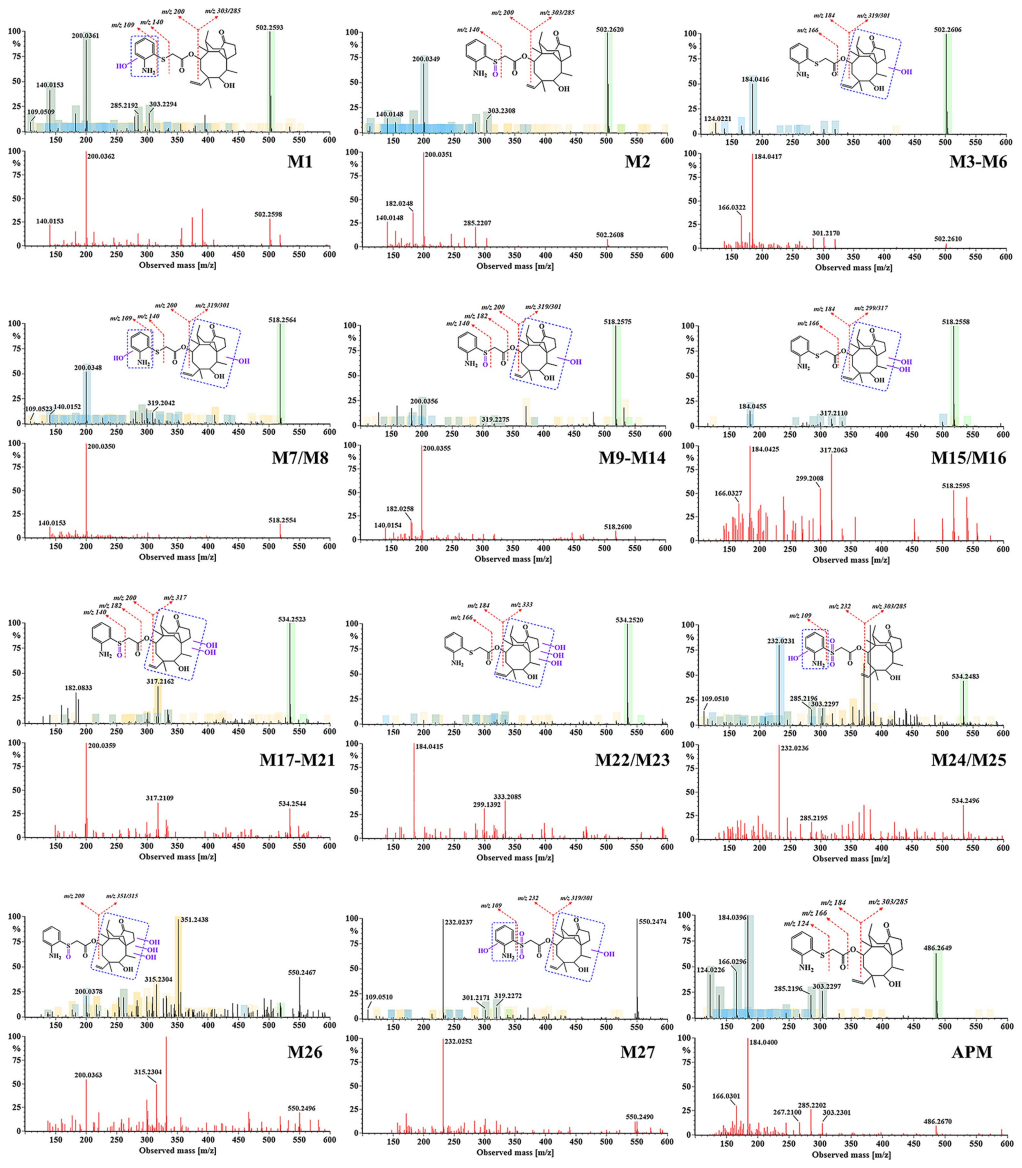
Mass spectra of APM metabolites and their fragment ion assignments (all spectra are shown in dual channels, upper, low energy; lower, high energy).

In the present work, we correlated mass spectra from the different energy channels of MS^E^ scans to complete an analysis of parent and fragment ions to calculate elemental composition and potential fragmentation pathways of the corresponding metabolites. We additionally utilized the differing LC retention times to distinguish isomers and their probable chemical structures. For ease of elucidation, all observed APM metabolites were categorized and isomers were grouped together. The accurate EICs of APM metabolites in samples from different animal species are displayed in Figure 4. The MS^E^ spectra and fragmentation patterns corresponding to these metabolites can be seen in Figure 5. Information regarding the *m/z* values, retention times, mass accuracy, and detection in various samples for all metabolites has been compiled in Table 2.

**Table 2:** Summary of all metabolites of APM *in vitro* and *in vivo*^a^.

ID	Description	Formula [M + H]^+^	Observed m/z	Retention time (min)	Mass error (mDa)	Mass error (ppm)	Major fragments	*In vitro*-microsome					*In vivo*-rat			*In vivo*-chicken	
								Human	Rat	Pig	Chicken	Dog	Plasma	Urine	Feces	Plasma	Feces
M0	APM	C_28_H_39_NO_4_S^+^	486.2675	13.7	0.2	0.5	184.04, 303.23, 285.22, 124.02, 267.21, 166.03	+	+	+	+	+	+	+	+	+	+
M1	APM + O	C_28_H_39_NO_5_S^+^	502.2624	9.1	0.3	0.5	109.05, 200.04, 140.02, 303.23	+	+	+	+	+	+	+	+	+	+
M2	APM-Sulfoxide	C_28_H_39_NO_5_S^+^	502.2620	11.9	−0.2	−0.3	200.04, 182.04, 140.02, 303.23,	+	+	+	+	+	+	ND	+	+	+
M3	APM + O	C_28_H_39_NO_5_S^+^	502.2644	10.0	2.3	4.5	184.04, 166.03, 301.22	+	+	ND	ND	+	ND	ND	ND	ND	ND
M4	APM + O	C_28_H_39_NO_5_S^+^	502.2607	10.6	−1.5	−2.9	184.04, 166.03, 319.23, 301.22	+	+	+	+	+	ND	+	ND	ND	+
M5	APM + O	C_28_H_39_NO_5_S^+^	502.2625	11.3	0.3	0.7	184.04, 124.02, 166.03, 301.22	+	+	+	+	+	+	+	+	+	+
M6	APM + O	C_28_H_39_NO_5_S^+^	502.2640	12.7	1.8	3.6	184.04, 301.22, 337.24	ND	+	ND	ND	+	ND	ND	ND	ND	ND
M7	APM + 2O	C_28_H_39_NO_6_S^+^	518.2575	6.1	0.4	0.8	200.04, 109.05, 319.23, 140.02	+	+	+	ND	+	ND	+	+	+	+
M8	APM + 2O	C_28_H_39_NO_6_S^+^	518.2582	6.9	1.1	2.1	200.04, 319.23, 109.05, 140.02	+	ND	+	ND	ND	+	+	+	+	+
M9	APM-Sulfoxide + O	C_28_H_39_NO_6_S^+^	518.2582	7.3	1.1	2.2	200.04, 301.22, 353.25	ND	ND	ND	ND	ND	ND	ND	+	+	+
M10	APM-Sulfoxide + O	C_28_H_39_NO_6_S^+^	518.2577	8.1	0.7	1.3	200.04, 182.03, 140.02, 301.22	+	+	+	ND	+	ND	+	ND	ND	ND
M11	APM-Sulfoxide + O	C_28_H_39_NO_6_S^+^	518.2574	8.6	0.3	0.7	200.04, 301.22, 162.91	+	+	+	ND	ND	ND	ND	ND	ND	ND
M12	APM-Sulfoxide + O	C_28_H_39_NO_6_S^+^	518.2571	8.9	0.0	0.0	200.04, 182.03, 140.02, 301.22	+	+	+	+	+	ND	+	+	+	+
M13	APM-Sulfoxide + O	C_28_H_39_NO_6_S^+^	518.2575	9.7	0.4	0.8	200.04, 182.03, 140.02, 319.23	+	ND	+	+	ND	+	+	+	+	+
M14	APM-Sulfoxide + O	C_28_H_39_NO_6_S^+^	518.2576	10.0	0.6	1.1	200.04, 182.03, 140.02, 301.22	+	ND	+	+	ND	ND	+	+	+	+
M15	APM + 2O	C_28_H_39_NO_6_S^+^	518.2558	9.5	−1.3	−2.5	184.04, 166.07, 317.21, 299.20	ND	+	ND	ND	+	ND	+	ND	ND	ND
M16	APM + 2O	C_28_H_39_NO_6_S^+^	518.2573	10.3	0.3	0.6	184.04, 335.22, 299.20, 155.1	+	+	+	ND	+	ND	+	ND	ND	ND
M17	APM-sulfoxide + 2O	C_28_H_39_NO_7_S^+^	534.2527	5.4	0.7	1.3	200.04, 317.21, 140.02, 295.09	+	+	+	ND	ND	ND	+	+	ND	+
M18	APM-sulfoxide + 2O	C_28_H_39_NO_7_S^+^	534.2532	5.7	1.2	2.3	200.04, 335.22, 317.21, 299.20	ND	ND	ND	ND	ND	ND	ND	+	+	+
M19	APM-sulfoxide + 2O	C_28_H_39_NO_7_S^+^	534.2509	7.2	−1.0	−1.9	182.03, 200.04, 317.21, 395.24	ND	ND	ND	ND	ND	ND	+	ND	ND	+
M20	APM-sulfoxide + 2O	C_28_H_39_NO_7_S^+^	534.2523	8.1	0.3	0.7	200.04, 317.21, 182.03	+	+	+	ND	ND	ND	ND	+	ND	+
M21	APM-sulfoxide + 2O	C_28_H_39_NO_7_S^+^	534.2517	7.7	−0.3	−0.5	200.04, 317.21, 166.03, 299.13	ND	ND	ND	ND	ND	ND	+	+	ND	+
M22	APM + 3O	C_28_H_39_NO_7_S^+^	534.2520	8.5	0.0	0.1	184.04, 299.13, 333.21	ND	ND	ND	ND	ND	ND	+	+	ND	+
M23	APM + 3O	C_28_H_39_NO_7_S^+^	534.2540	9.5	2.1	3.9	184.04, 333.21, 315.20, 180.10	ND	ND	ND	ND	ND	ND	ND	+	ND	ND
M24	APM-sulfone + O	C_28_H_39_NO_7_S^+^	534.2522	10.2	0.2	0.4	109.05, 232.02, 303.23, 285.22	+	ND	ND	ND	ND	ND	ND	+	+	+
M25	APM-sulfone + O	C_28_H_39_NO_7_S^+^	534.2509	10.9	−0.1	−0.2	109.05, 232.02, 303.23, 267.21	ND	ND	ND	ND	ND	ND	ND	+	ND	ND
M26	APM-sulfoxide + 3O	C_28_H_39_NO_8_S^+^	550.2467	7.5	−0.2	−0.4	351.24, 200.04, 315.20, 331.08	ND	ND	ND	ND	ND	ND	ND	+	ND	ND
M27	APM-sulfone + 2O	C_28_H_39_NO_8_S^+^	550.2474	8.0	0.5	0.9	109.05, 232.02, 301.22, 319.23	ND	ND	ND	ND	ND	ND	ND	+	ND	ND

+, detected; ND, undetected.

### Metabolites M1–M6

Metabolites M1 to M6 possessed retention times of 9.1–12.7 min and all produced quasi-molecular ion peaks at *m/z* 502.26 [M + H]^+^ with associated mass errors below 5 ppm. Their calculated elemental composition, C_28_H_39_NO_5_S^+^, suggested the addition of one *O* atom (16 Da) compared to APM, indicating these were oxidation or hydroxylation products. However, the varying retention times also suggested that they are isomers. In particular, M1 and M2 shared the same fragment ions *m/z* 303 and 285 suggesting that the parent mutilin nucleus remained unchanged and the metabolic reactions occurred at the side chains. However, M1 has an additional fragment ion at *m/z* 109 corresponding exactly to -OH addition to the anilino group. Given the structural features of APM, we favor the hypothesis that the M1 hydroxylation occurred on the more reactive amino group. The M2 *m/z* 140 fragment ion suggested that it is likely a metabolite of *S* oxidation, i.e., sulfoxidized amphenmulin. Both M1 and M2 were detected in all species investigated in this study.

The M3-M6 ions possessed the same *m/z* 184 side chain fragment ion as the parent drug suggesting that their hydroxylation sites occurred at the mutilin nucellus. This was supported by the fragment ions at *m/z* 319 and 301. The European Medicines Agency (EMA) previously identified 8α-OH tiamulin as its primary metabolite and designated it as a residue marker in pigs and chickens (32). Other studies have confirmed that the primary hydroxylation sites on the pleuromutilin nucleus of tiamulin are at the 2β and 8α positions (23, 24). Similarly, for valnemulin and retapamulin, hydroxylation or oxidation at the mutilin nucleus is a common metabolic characteristic of these compounds (33, 34). This provides important reference for our study. However, due to the complex and diversity of the metabolic sites on the pleuromutilin nucleus, as well as the varying abundance of these metabolites in samples from different species, we are not yet able to identify the specific hydroxylation sites.

### Metabolites M7–M16

Metabolites M7 to M16 were chromatographically separated between 6.1–10.3 min and exhibited the same [M + H]^+^ ions at *m/z* 518.26 (C_28_H_39_NO_6_S^+^) and this was 32 Da higher than the quasi-molecular ion peak of APM. This suggested APM double hydroxylation or oxidation products. M7-M8 shared the same fragment ions at m/z 200 and 109 with M1, indicating identical side chain modifications. However, the fragment ions at m/z 319 and 301 were exactly 16 Da greater than mutilin fragments m/z 303 and 285, suggesting that they are hydroxylated from different sites on the mutilin rings of M1, which led to their separation and identification due to the relative polarity in the chromatogram. In contrast, M9-M14 lacked the precise *m/z* 109 fragment ion inferring that they are products of further hydroxylation on the parent nucleus based on M2. The MS/MS spectra of M15 and M16 exhibited typical APM side chain fragment ions at *m/z* 184 and 166 while their fragment ions at *m/z* 299 and 317 were 32 Da more than the parent drug fragments at *m/z* 267 and 285. This confirmed their identities as di-hydroxylated metabolites of the mutilin nucleus.

### Metabolites M17–M25

The M17-M25 (*m/z* 534.25, C_28_H_39_NO_7_S^+^) fragments exhibited a mass increment of 48 Da compared to APM and was consistent with the addition of three *O* atoms. This suggests that they are likely products of further hydroxylation or oxidation of M7-M16. In particular, M17-M21 were eluted and separated within 5.4–8.1 min and shared the same fragment ions at *m/z* 317, 299, 200 and 140 that were 32 and 16 Da greater than the APM parent nucleus and side chain fragments, respectively. This indicated that they are metabolites of sulfur oxidation on the side-chain along with the dihydroxylation of the mutilin rings. Metabolites M22 and M23 had retention times of 8.5 and 9.5 min, respectively. They possessed characteristic fragment ions at *m/z* 184 and 166 and their fragments at *m/z* 333 and 315 were exactly 48 Da greater than the parent drug fragments at *m/z* 285 and 267. This indicated that they were products of triple hydroxylation at the mutilin nucleus and lacked side-chain modifications. M24 and M25 were eluted ~10.2 and 10.9 min, respectively and their fragments at *m/z* 303 and 285 indicated mutilin rings were not modified while fragments *m/z* 232 and 109 demonstrated an addition of 3 *O* atoms (48 Da) to the side chain. M24 and M25 are highly likely to be the products of the hydroxylation of aniline groups at different positions with simultaneous double oxidation of the sulfur atom (sulfonation).

### Metabolites M26, M27

Metabolites M26 and M27 possessed retention times of 7.5 and 8.0 min, respectively and both were found only in rat feces. They produced a precursor ion at *m/z* 550.25 corresponding to the elemental composition of C_28_H_39_NO_8_S^+^ that was 64 Da greater than that of the parent compound. This suggested that M17-M25 can still be further hydroxylated or oxidized *in vivo*. The M26 fragment ions *m/z* 351 and 315 were 48 Da greater than the parent drug fragments *m/z* 303 and 267 and when combined with the fragment *m/z* 200 indicated that M26 was most likely a metabolite of M2 after the triple hydroxylation of the mutilin nucleus. The metabolite M27 with product ions at *m/z* 319 and 301, indicated a single hydroxylation on its parent nucleus. Additionally, the ion at *m/z* 232 suggests an additional 3 *O* atoms on its side chain so we can infer that M27 is a product of hydroxylation of the M24/M25 mutilin portion.

### Metabolic pathways of APM

**Figure 6:**
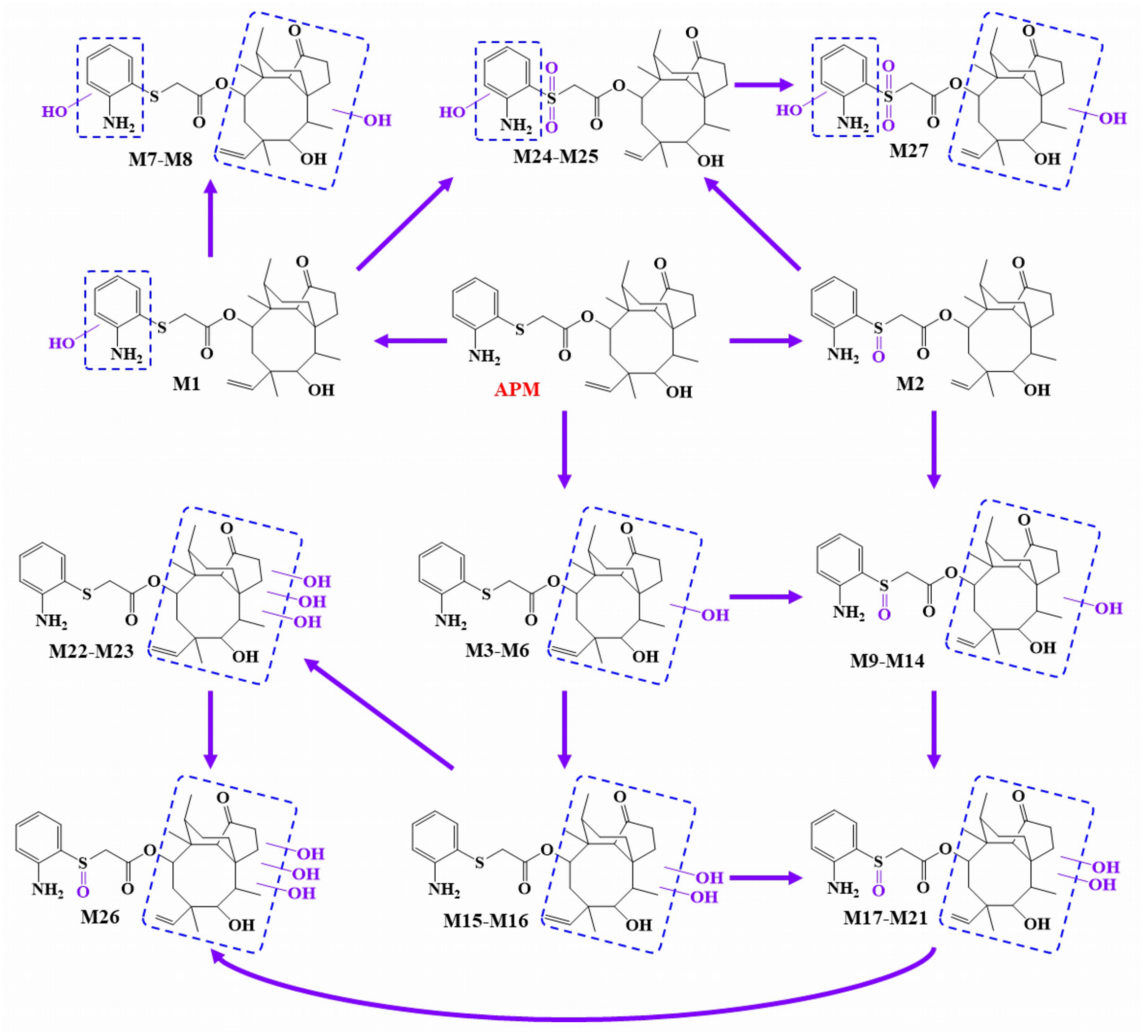
Proposed APM metabolic pathways *in vivo* and *in vitro*.

We integrated the metabolites from different species *in vivo* and *in vitro* and obtained the primary APM metabolic pathways (Figure 6). These included oxidation of the *S* atom in the side chain, hydroxylation of the aniline moiety, along with mono-and multi-level hydroxylation of the pleuromutilin nucleus. No phase II metabolites were detected.

### Comparative metabolism of APM in liver microsomes of different animals

**Figure 7:**
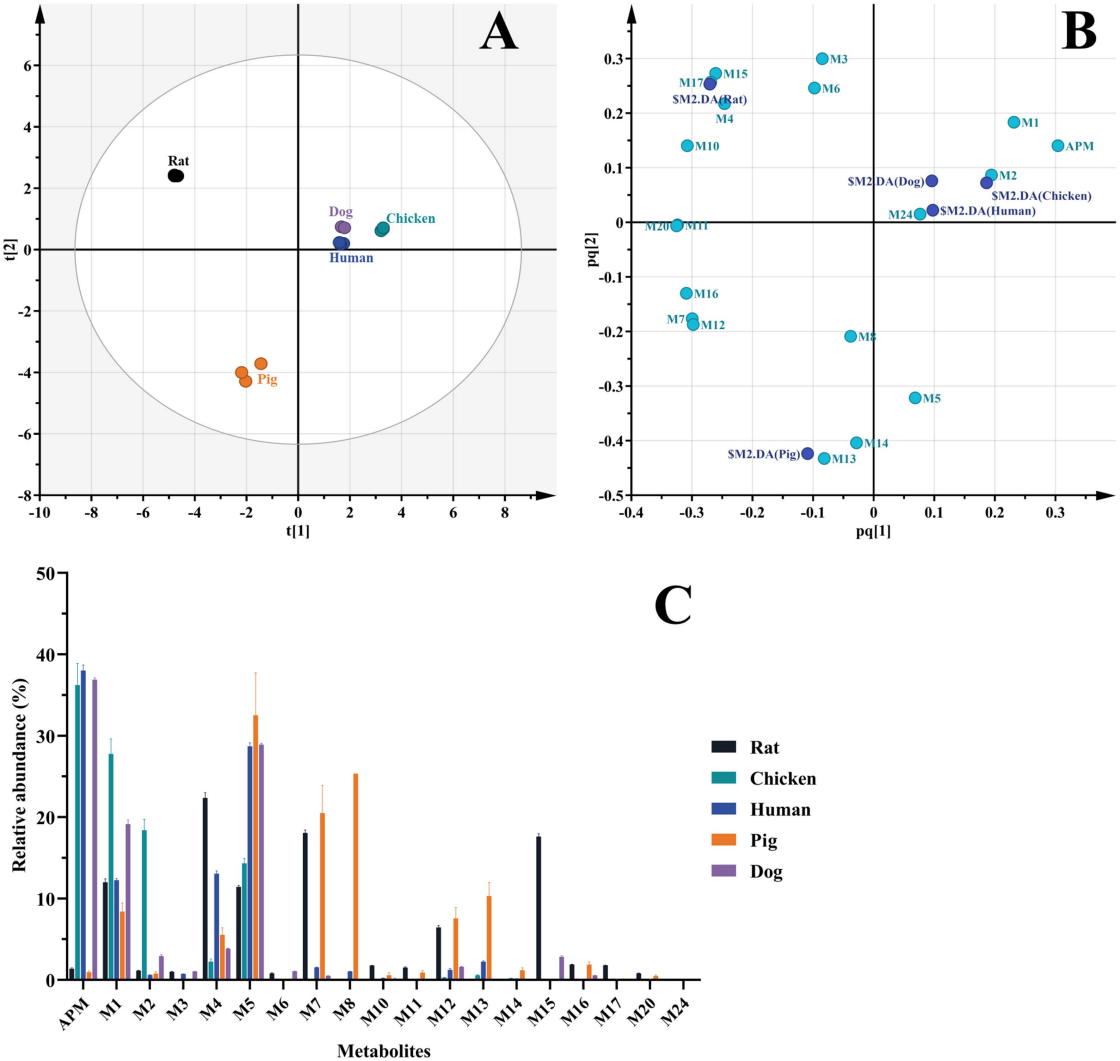
Comparison of APM metabolites *in vitro*. **(A)** OPLS-DA score plot of metabolites in liver microsomes of different animals; **(B)** OPLS-DA loading scatter plot; **(C)** Relative abundance of APM metabolites in liver microsomes.

The liver microsomal incubation system *in vitro* generated 14 rat, 16 human, 14 pig, 12 dog, and 7 chicken metabolites. Again, no phase II metabolites found. As illustrated in the score scatter plot (Figure 7A), metabolite samples from different animals display clear clustering and separation, and as seen in Figure 7B, the corresponding loading scatter plot highlights the distribution of various metabolites (light blue labels) in relation to the different animal groups (dark blue labels). Furthermore, due to the limitations of Q/TOF-MS at full scan, we were unable to accurately quantify each metabolite. By comparing the peak areas of individual metabolites in the samples to the sum of the peak areas of all identified components, we obtained their relative abundance. This semi-quantitative measure is based on estimated signal intensities under the assumption of equal ionization efficiency, and was used as a reference to identify the primary metabolites. The interspecies distribution and relative abundance of the detected metabolites are presented in Figure 7C.

In particular, metabolites M1, M2 (APM-sulfoxide), M4, M5 and M12 were detected in the liver microsomal system of all 5 animals. In rats, the primary metabolites were M4, M7 and M15; in humans, M5, M4 and M1; in pigs, M5, M8 and M7 and in beagle dogs, M5, M1 and M4. The transformation of APM in the liver microsomes of these animals was primarily through mono-and multi-level hydroxylation of the pleuromutilin nucleus. The primary metabolites for chicken microsomes were M1, M2, and M5 and indicated that APM was metabolized via side-chain *S* oxidation and aniline hydroxylation. This scheme differed from those of the other animal microsomes we examined as well as from the APM analogs tiamulin and lefamulin, which primarily undergo hydroxylation at the pleuromutilin nucleus (21, 23, 32). Additionally, based on the proportion of remaining parent drug in the samples, APM was metabolized to a greater extent in rat and pig microsomes with a broader distribution of metabolite proportions than for human, chicken and beagle dog microsomes. These results indicated overall species differences for *in vitro* APM metabolism in liver microsomes.

### Metabolism of APM in rats and chickens

**Figure 8:**
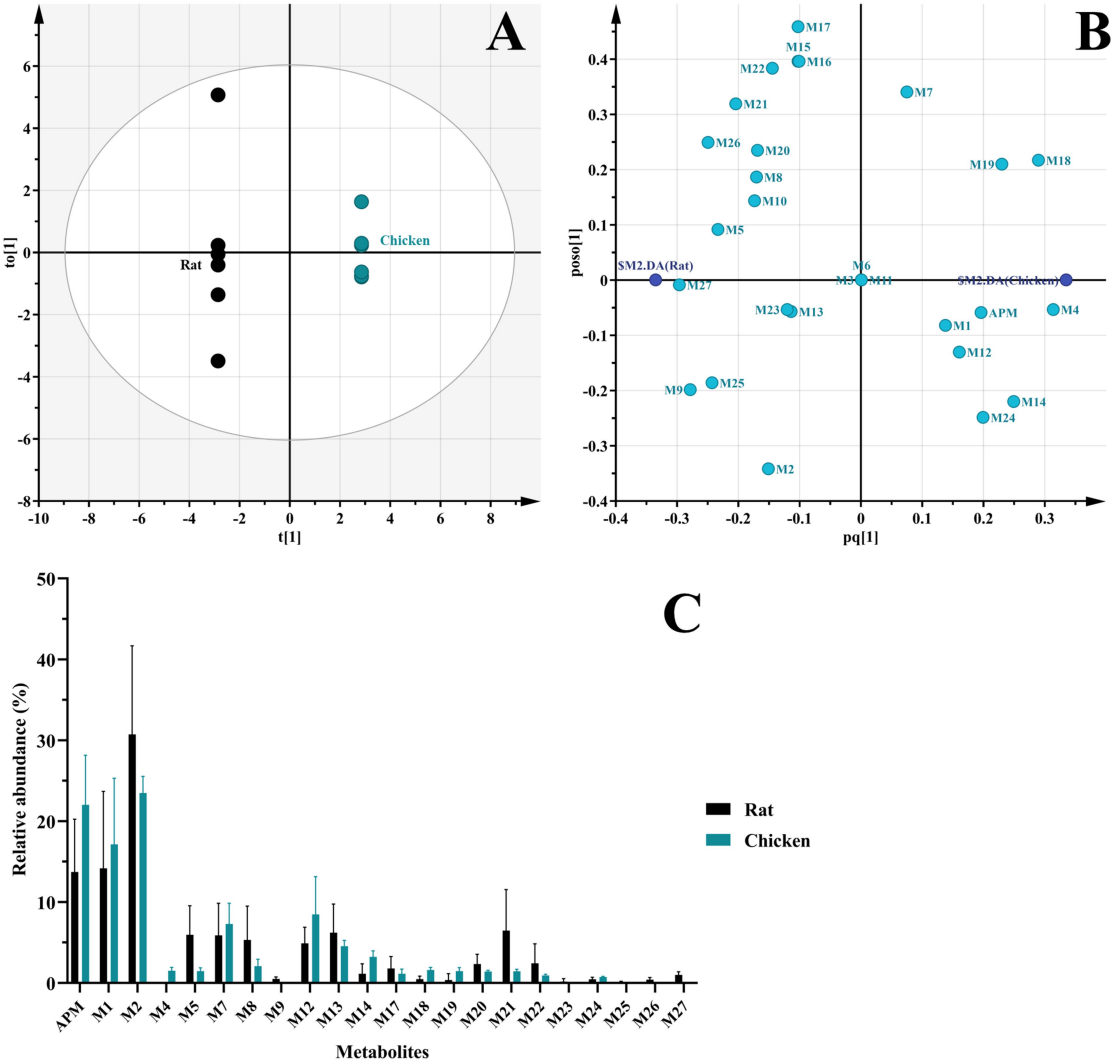
Comparison of APM metabolites *in vivo*. **(A)** OPLS-DA score plot of metabolites in fecal and urinary samples from rats and chickens; **(B)** OPLS-DA loading scatter plot; **(C)** Relative abundance of APM metabolites *in vivo*.

*In vivo* samples from rats and chickens generated 24 and 17 APM metabolites, respectively. The OPLS-DA score plot reveals two distinct clusters corresponding to the two species (Figure 8A), indicating significant separation of the samples. Meanwhile, the scatter distribution on either side of the horizontal axis in the loading plot reflects the metabolite differences between the rat and chicken groups (Figure 8B). In contrast to liver microsomes, APM was more extensively metabolized *in vivo* that was characterized primarily by multi-level oxidation or hydroxylation reactions. Again, no phase II metabolites were observed. These metabolites were primarily concentrated in feces samples, urine (in rats) and the lowest abundance were plasma. This indicated that APM was primarily excreted via feces. And similar to other pleuromutilin derivatives, APM also exhibits high lipophilicity and extensive tissue distribution (17, 35, 36), this process may be the cause of the relatively lower levels of metabolites we detected in plasma samples. And the relative abundance of *in vivo* metabolites indicated that APM is highly metabolized in rats and the multi-level metabolites like M25, M26, and M27 were not found in the *in vivo* chicken samples (Figure 8C). Furthermore, in contrast to the primary metabolic pathways of tiamulin involving 2β-and 8α-hydroxylation and *N*-deethylation (23), APM shows less pronounced mono-hydroxylation metabolism in the mutilin portion *in vivo* since we detected only trace amounts of metabolites M4 and M5. On the other hand, several polyhydroxylated metabolites were exclusively observed *in vivo* but not in liver microsomes, likely due to extrahepatic metabolism, sequential biotransformation steps, and the longer exposure time in systemic circulation. These factors are not fully replicated in the microsomal system, which may explain the absence of certain metabolites *in vitro*. The overall *in vivo* results indicated that M2 (APM-sulfoxide) was a major metabolite in both rats and chickens and was consistent with prior results of valnemulin metabolism in chickens (26), where the *S* atom on the side chain is recognized as a significant reactive site for this class of drugs. Secondly, metabolite M1 was also found in considerable proportions both the chickens and rats and this corresponded to the reactive aniline group at the end of the side chain. These findings were consistent with the SOM predictions made by SMARTCyp, yet the precise metabolic sites cannot be determined at present.

## Conclusion

This study employed the high-resolution data acquisition and processing techniques of UHPLC-Q/TOF to rapidly and reliably analyze and screen a total of 27 Phase I metabolites of APM (18 from liver microsomes, 24 from rats and chickens), while no Phase II conjugates detected. The metabolic pathways of APM were revealed to be primarily hydroxylation and oxidation of the side chain (sulfoxidation) and hydroxylation of the mutilin nucleus. The primary metabolite of APM in rats and chickens was APM-sulfoxide and hydroxy-APM, which should be prioritized in future studies.

Pleuromutilin derivatives present a current and active field of research due to their excellent antimicrobial properties. Our findings complement the theoretical gaps in the early studies of APM as a new drug. This data can assist subsequent studies on the evaluation of the bioactivities and potential toxicities of key metabolites, as well as on residue elimination in food-producing animals. In particular, the comprehensive characterization of major metabolites will support further toxicological risk assessment and aid in the selection of appropriate marker residues.

## Data Availability

The original contributions presented in the study are included in the article/supplementary material, further inquiries can be directed to the corresponding author.

## Footnotes

^1^ https://smartcyp.sund.ku.dk/mol_to_som
